# The PI3K inhibitor pictilisib and the multikinase inhibitors pazopanib and sorafenib have an impact on Rac1 level and migration of medulloblastoma in vitro

**DOI:** 10.1111/jcmm.17604

**Published:** 2022-11-15

**Authors:** Leonie F. Schoen, Rogerio B. Craveiro, Torsten Pietsch, Thomas Moritz, Anja Troeger, Silvia Jordans, Dagmar Dilloo

**Affiliations:** ^1^ Department of Pediatric Hematology and Oncology, Center for Pediatrics University Hospital Bonn Bonn Germany; ^2^ Department of Orthodontics University Hospital RWTH Aachen Aachen Germany; ^3^ Department of Neuropathology University Hospital Bonn Bonn Germany; ^4^ Institute of Experimental Hematology Hannover Medical School Hannover Germany; ^5^ Department of Pediatric Hematology, Oncology and Stem Cell Transplantation University Hospital Regensburg Regensburg Germany

**Keywords:** actin cytoskeleton, cell migration, ERK/MAPK, medulloblastoma, PI3K, Rac1

## Abstract

Metastatic disease is the leading cause of death in children suffering from medulloblastoma and a major treatment challenge. The evidence of leptomeningeal dissemination defines the most aggressive tumours and is associated with increased mortality; thus, inhibition of migration as a factor involved in the process of metastatic disease is fundamental for the treatment and prevention of metastatic dissemination. Targeting the small Rho GTPases Rac1 has been shown to effectively impair medulloblastoma cell migration in vitro. Yet clinically applicable selective Rac1 inhibitors are still lacking. In view of the pertinent oncogenic role of the PI3K signalling cascade and tyrosine kinase‐mediated signalling pathways in medulloblastoma, we explored clinically available targeted therapeutics to this effect. Here, we show that Rac1 is expressed in both the cytoplasm and nucleus in the medulloblastoma cell lines Daoy and MEB‐Med‐8A representative of two high risk medulloblastoma entities. We demonstrate that activated Rac1 is subject to substantial downmodulation following administration of the clinically available inhibitor of the PI3K pathway Pictilisib (GDC‐0941) and the multityrosine kinase inhibitors Pazopanib and Sorafenib. The application of those drugs was associated with reduced mobility of the medulloblastoma cells and alterations of the actin skeleton. Of note, PI3K inhibition reveals the strongest anti‐migratory effect in Daoy cells. Thus, our in vitro observations provide new insights into different strategies of blocking Rac1 and inhibiting migration in medulloblastoma employing clinically available agents paving the way for confirmatory studies in in vivo models.

## 1 INTRODUCTION

Medulloblastoma is the most common malignant brain tumour in children. Despite ongoing optimization of standard therapy, the prognosis for high‐risk cases is still poor.^1^, ^2^ The current WHO classification divides medulloblastoma into different molecularly defined types each associated with a distinct risk profile.^3^ Clinically, disseminated disease is present in up to one‐third of medulloblastoma patients at diagnosis and has a high negative impact on survival representing one of the major treatment challenges.^2^, ^4^, ^5^, ^6^ Thus, novel therapeutic strategies geared towards inhibition of local and metastatic spread are warranted to improve prognosis.^2^, ^7^

Several signalling pathways have been shown to be involved in tumour dissemination in medulloblastoma. The platelet‐derived growth factor receptor (PDGFR) overexpressed in metastatic medulloblastoma was found to be one of the key modulators of metastasis.^8^, ^9^, ^10^, ^11^ Activation of upstream tyrosine kinase receptors drives oncogenic processes in medulloblastoma via the Ras/MAPK pathway as well as PI3K signalling.^12^, ^13^, ^14^, ^15^ Downstream, the MAPK‐ and PI3K‐pathways influence the activity of Rho GTPases. They represent a family of small signalling G proteins that orchestrate conformational changes of the actin cytoskeleton modulating cellular polarity and motility. Rho GTPases cycle between an active GTP‐bound and an inactive GDP‐bound conformation with guanine exchange factors (GEF) supporting the activation state by deactivation of GTPase‐activating proteins (GAP). Furthermore, guanine nucleotide dissociation inhibitors (GDI) stabilize Rho GTPases in an inactive state.^16^, ^17^ There is abundant evidence for the central role of the Rho GTPase Rac1 for tumour invasiveness and metastatic growth. Rac1 has also been attributed a central role in epithelial to mesenchymal transition (EMT) enhancing the migratory capacity of tumour cells with a negative impact on prognosis.^17^, ^18^, ^19^ Rac1 expression has been confirmed by immunohistochemistry in a series of medulloblastoma specimens and has been shown to act as a central effector of migration in medulloblastoma models.^9^, ^18^, ^20^, ^21^, ^22^ While genetic silencing or chemical inhibition have been successfully employed so far in tumour models, no pharmacological Rac1‐specific inhibitors are currently available for clinical use.^18^, ^21^, ^22^ Therefore, the application of clinically approved drugs that either interfere with upstream regulators of Rac1 activation or block downstream effectors represent a potential therapeutic option worth exploring.

We have previously demonstrated that the multikinase inhibitors (MKI) Pazopanib and Sorafenib targeting the PDGFR among other tyrosine kinase receptors and the direct PI3K‐inhibitor Pictilisib (GDC‐0941) exhibit potent anti‐neoplastic capacities in orthotopic xenograft models of medulloblastoma including anti‐migratory effects in vitro.^23^, ^24^ Thus, the three drugs target signalling elements upstream of Rac1 and show acceptable tolerability in clinical studies in addition to anti‐neoplastic activity in different tumour types including advanced renal cell carcinoma, hepatocellular carcinoma and solid tumours.^25^, ^26^, ^27^ In the present report, we demonstrate that both MKI and the PI3K inhibitor reduce levels of activated Rac1 in two medulloblastoma cell lines representing two molecularly defined medulloblastoma entities with high metastatic potential. The cell line MEB‐Med‐8A has been derived from anon‐WNT/non‐SHHMB (group 3) and bears a MYC amplification which has been shown to promote migration and metastasis in medulloblastoma.^28^, ^29^ The cell line Daoy displays markers of SHH activation and carries a mutation of the TP53 suppressor gene.^29^, ^30^ We describe here distinct modulation of the actin cytoskeleton and inhibition of migration in these two medulloblastoma cell lines through targeting critical signalling pathways upstream of Rac1.

## 2 MATERIAL AND METHODS

### 2.1 Reagents and antibodies

Pictilisib (GDC‐0941) applied at 1 μM, Pazopanib (GW786034B) applied 15 μM and Sorafenib (BAY 43–9006) applied at 10 μM were obtained from LC Laboratories and dissolved in cell culture grade DMSO (Sigma Aldrich). All antibodies used in this study are given in Table S1 (supplementary files). Alexa Flour® 555 coupled Rhodamine Phalloidin for actin staining was purchased from Invitrogen, Thermofisher.

### 2.2 Cell culture

The medulloblastoma cell line Daoy (HTB 186) was obtained from ATCC and the medulloblastoma cell line MEB‐Med‐8A was kindly provided by Prof. T. Pietsch, University of Bonn. Both were maintained in complete medium comprising Dulbecco's Modified Eagle Medium (DMEM, Invitrogen) including L‐glutamine and supplemented with 1 mM sodium pyruvate (PAA), 100 U/ml penicillin (Invitrogen), 100 μl/ml streptomycin (Invitrogen) and 10% foetal bovine serum (FBS, Invitrogen) under standard culture conditions.

### 2.3 Immunofluorescence microscopy

Daoy (2 × 10^4^) and MEB‐Med‐8A (4 × 10^4^) cells were seeded in 12‐well culture dishes containing sterilized coverslips and allowed to adhere overnight in complete medium. Cells for scratch assays were seeded on glass coverslips (Rac1 staining) or in 12‐wells (actin stains) to reach about 90% of confluency. Then, monolayers were scratch wounded using a pipette tip and wells were washed once with prewarmed PBS before applying new medium containing inhibitors in the above‐mentioned concentrations.

Inhibitor treatment was performed for indicated time intervals and cells were fixed afterwards with 4% paraformaldehyde for 10 min at 37°C. Following 5 min permeabilization (0,2% Triton X‐100, Boehringer Mannheim) and 60 min blocking with 3% BSA in PBS, cells were stained with primary antibody overnight in a moisturized chamber at 4°C. After washing with PBS, secondary antibodies were applied for 90 min at RT. Rhodamine Phalloidin (Invitrogen, Thermofisher) was added for 20 min (scratch assays) or in combination with the secondary antibody (Rac1/actin counterstain). Cells were embedded in Prolong Glass antifade solution including DAPI (Invitrogen) to stain nuclei. Immunofluorescence microscopy was performed with a Nikon Eclipse TiS inverted microscope equipped with a CCD monochrome camera DS 2 M. Images were analysed with Fiji imaging software (ImageJ, 1.53c/NIH). Confocal microscopy was performed with the Nikon A1 LFOV in Galvano Scan mode (for 488 nm and 561 nm laserlines) or PMT (405 nm laserline) at a pinhole setting of 1 AU and a dimension of 1024 × 1024 pixels. Laser power, PM detector gains and offset were set with DMSO samples and kept constant for inhibitor‐treated samples of the same experimental batch to ensure comparability. Z‐stacks from top to bottom of the cells were taken in 0.3 μm steps and extended focus pictures were prepared in average intensity Z‐projection mode (ImageJ, 1.53c/NIH).

### 2.4 Analysis of cell migration after scratch wounding

The in vitro scratch assay was performed as described by Liang et al.^31^. Briefly, Daoy and MEB‐Med‐8A cells were plated in 12‐well cell culture dishes and allowed to adhere and spread for 12 h at 37°C. The confluent monolayer was scratched in a straight line with a p200 pipette tip for Daoy or a p100 pipette tip for MEB‐Med‐8A. Cellular debris was washed off and the cells were incubated with 1 μM Pictilisib (GDC‐0941), 15 μM Pazopanib or 10 μM Sorafenib. The migration of the cells into the scratch was documented at the same area every 6 h of treatment using 10× magnification (Nikon Eclipse TiS inverted microscope equipped to a CCD monochrome camera DS 2 M). The scratch closure was analysed using NIS‐Elements Imaging Software by assigning cell‐free areas as ROI and comparing decrease of wound area between untreated and treated conditions after indicated time intervals.

### 2.5 Determination of cell viability and cell death effects

Medulloblastoma cell lines Daoy (3 × 10^5^) or MEB‐Med‐8A (7 × 10^5^) were seeded in 6‐well cell culture dishes in complete medium. After overnight culture, cells were treated with inhibitors for 6, 12 or 24 h. Supernatants and trypsinized cells were collected and combined after the indicated time intervals, resuspended in an equal volume of PBS and stained with Propidium‐Iodide (Miltenyi Biotech). The number of viable versus dead cells was analysed by Flow Cytometry (Navios, Beckman Coulter) by counting cells for 120 s in the same volume and at constant speed.

### 2.6 GTP‐bound Rac1 pull‐down assay

A Rac1 Activation Magnetic Beads Pulldown Assay was performed following the manufacturer's instructions (Merck Millipore). Briefly, cells were cultured on cell culture dishes in complete medium. Cells were treated with inhibitors for 24 h. After collection and centrifugation of floating cells, the monolayers were washed once with ice‐cold PBS, scraped off the dish in 800 μl of the provided lysis buffer including phosphatase and protease inhibitors (Roche), and combined with the floating cells' fraction. After pelleting cell debris at 4°C for 10 min, the supernatant was measured for protein content using protein Assay (BioRad). For assessment of total Rac1 protein, 25 μg of lysates were separated by SDS‐PAGE while for GTP‐bound Rac1 samples were diluted to 1 mg/ml in lysis buffer and 1 mg of total protein was incubated with 10 μl PAK‐PBD for 45 min at 4°C. Beads were magnetically collected, repeatedly washed in lysis buffer, centrifuged and resuspended in 10 μl of 2× Laemmli sample buffer (Bio‐Rad).

### 2.7 Subcellular fractionation

Subcellular fractions were prepared following the method described in Tong et al.^32^ Briefly, cells were grown in 10 cm culture dishes for 48 h, washed twice with cold PBS and scraped off the plates in 500 μl homogenization buffer including phosphate and protease inhibitor mix (Roche). Homogenates were passed through a 23‐gauge needle 20 times on ice and nuclei were separated from cytoplasmatic and membrane contents by centrifugation at 200 × *g* for 10 min at 4°C. The supernatants were collected as ‘cytosolic fraction’, while the pellet contained the ‘nuclear fraction’. Cytosolic fractions were centrifuged at 14,000 × *g* for 10 min to clear the fraction from nuclear remains. Nuclear fractions were resuspended in 200 μl of homogenization buffer and again centrifuged at 200 × *g* for 10 min at 4°C to remove remains of the cytoplasm and membranes. This washing step was repeated 3 times. The last nuclear pellet was resuspended in 100 μl of RIPA buffer (Merck Millipore, incl. inhibitors). As nuclear fractions contained a high amount of DNA, samples were subjected to ultrasound treatment for 5 min to fragment DNA strands. Equal protein amounts were loaded on SDS‐PAGE according to protein determination (Precision Red Advanced Protein Assay Reagent, Cytoskeleton Inc.).

### 2.8 Immunoblotting and densitometry

**FIGURE 1:**
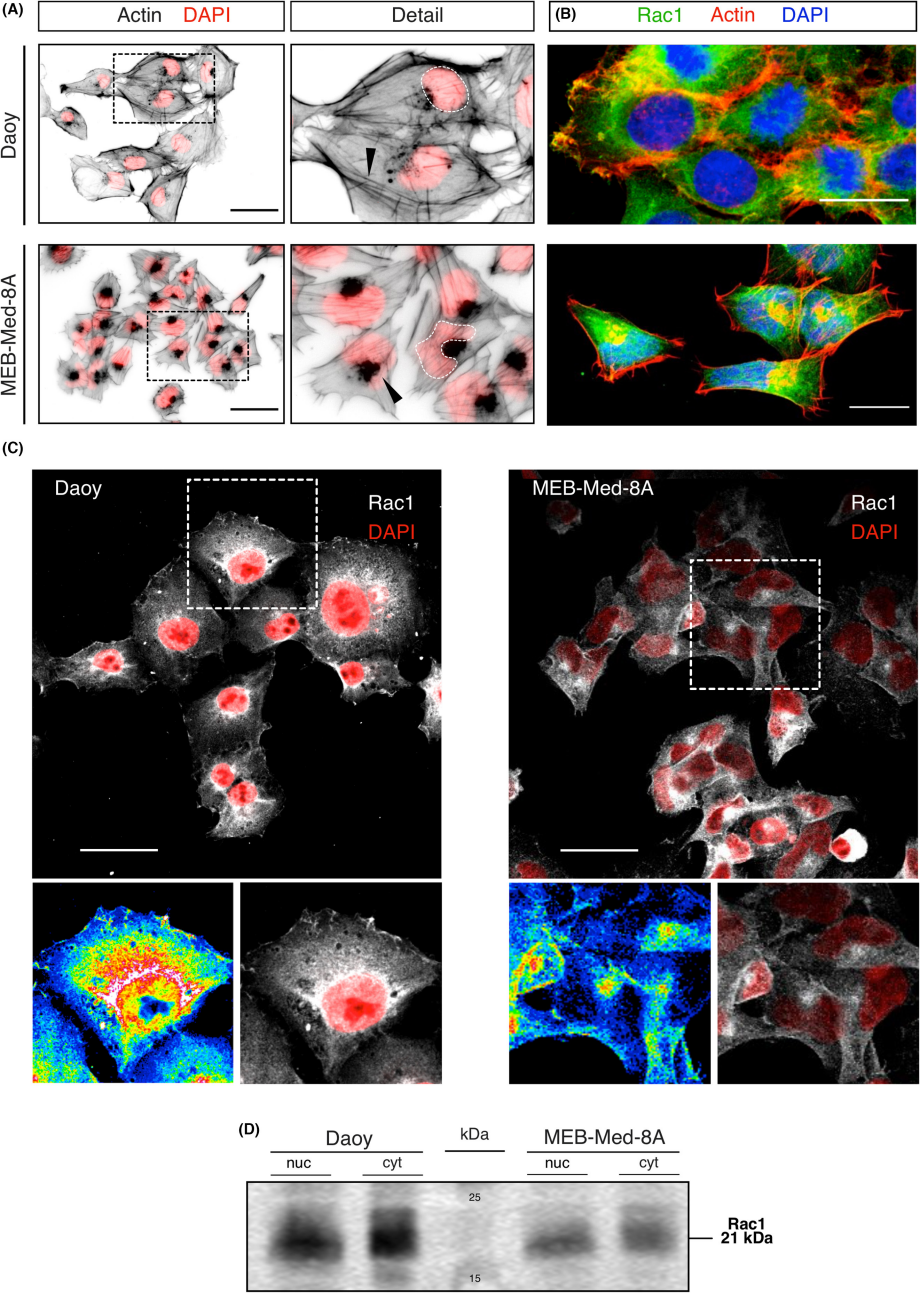
Medulloblastoma cells of different subgroups reveal distinct actin cytoskeletal features, nuclear shape, and differential distribution of Rac1. (A) Actin skeleton of Daoy and MEB‐Med‐8A cells was stained with Alexa Fluor‐555 coupled phalloidin 24 h after seeding and imaged by fluorescence microscopy (40x objective). Grey scale images were inverted to show phalloidin‐staining (actin) in black and merged with RGB images showing nuclei (DAPI counterstain) in red. Dashed white lines outline nuclear shapes displaying a characteristic deformation of the nucleus in MEB‐Med‐8A cells. Actin cytoskeleton was arranged in a prominent circular structure underlying the cellular cortex in Daoy cells or in distinct perinuclear accumulations in MEB‐Med‐8A cells (black arrowheads, detail view) situated in the characteristic nuclear indentation. Pictures are representative of three independent experiments. Scales indicate 50 μm. (B) Microfilaments (actin, red), nucleus (DAPI, blue) and Rac1 (green) were stained in medulloblastoma cell lines and Z‐stacks were prepared by means of confocal laser‐scanning microscopy with a 40x water immersion objective (NA 1.15). The extended focus pictures show subcellular distribution of Rac1 with respect to actin filaments. Rac1 is seen in the cytoplasm in both cell lines and additionally localizes to the cortical network at the plasma membrane in Daoy cells, while in MEB‐Med‐8A cells Rac1 is located to the microfilament accumulation in close vicinity to the nucleus but does not seem to be present at high concentrations at the cell boundary. Scales indicate 20 μm. (C) Daoy and MEB‐Med‐8A cells were stained for Rac1 (grayscale) and DAPI (red) and imaged by confocal laser‐scanning microscopy as described in (B). Extended focus pictures show the subcellular distribution of Rac1 in the two different cell lines with respect to the nucleus and cytoplasm. Detailed views (inserts) in grayscale and 16‐colour lookup tables (LUT) illustrate that the major proportion of Rac1 is in a ring like structure around the nucleus and thinned out towards the cellular border. In MEB‐Med‐8A cells the maximum signal of Rac1 was captured in an aggregation equivalent to the actin accumulation and situated close to the nucleus or within the nuclear indentation characteristic of MM8A. Scales indicate 50 μm. (D) Subcellular fractions of medulloblastoma cells were analysed by immunoblot to assess the presence of Rac1 in the cytoplasmatic and nuclear compartment of both cell lines. Either fraction shows positive signals for Rac1 upon detection using a Rac1 specific antibody (BD Biosciences). The expected molecular weight (21 kDa) is confirmed by the molecular weight marker shown in the middle lane (kDa). Results are representative of three independent experiments.

Separated proteins from SDS‐PAGE were transferred onto nitrocellulose membranes by semi‐dry blotting at 25 V for 30 min (Bio‐Rad). The membranes were blocked for 1 h at RT in 1 × Tris‐buffered saline containing 0.1% Tween‐20 (TBST) supplemented with 5% BSA. Primary antibody incubation was carried out overnight at 4°C and followed by incubation with the respective secondary antibody for 1 h at room temperature. Immunoreactivity was detected by chemiluminescence (PIERCE ECL western blotting substrate, Thermofisher) and quantified by ChemiDoc XRS Imaging System (Bio‐Rad). Blots from Figure 1D were analysed with the SpectraMax i3X (Molecular Devices) equipped with a ScanLater module in high resolution scan. Densitometry of Western blots was performed using ImageJ 1.53c.

### 2.9 Statistical analysis

The two‐sided Student's t‐test (Graph Pad Prism) was applied to determine statistical significance of difference between groups. *p* < 0.05 (*) was considered as statistically significant. Values stated within the text and figures represent mean ± standard deviation.

## 3 RESULTS

### 3.1 Daoy and MEB‐Med‐8A exhibit different morphology of the actin skeleton

The two medulloblastoma cell lines Daoy and MEB‐Med‐8A representative of different molecular MB types with high metastatic potential present with a different morphology of the actin cytoskeleton after phalloidin staining (Figure 1A). Daoy cells displayed a voluminous cortical actin network framing the cytoplasmatic border. MEB‐Med‐8A cells were overall smaller and appeared more compact in direct comparison with Daoy cells. They exhibited prominent stress fibres, and the membranes showed less actin‐rich protrusions. The nuclei of MEB‐Med‐8A cells were usually kidney‐shaped, sometimes also highly deformed with aggregates of actin located next to the indentation of the nucleus. Rac1 is ubiquitously expressed in the human body and locates to both, the cytoplasm and the nucleus.^33^ In Daoy cells, Rac1 accumulated in a circular formation around the nucleus with gradual dissipation into the cytoplasm towards the plasma membrane where accentuated Rac1 staining was shown. In MEB‐Med‐8A cells, compact Rac1 accumulations were found in the nuclear indentation localizing in the same place as the actin conglomeration and additionally dispersed throughout the cytoplasm (Figure 1B). In keeping with the Western blot analysis of cytoplasmatic and nuclear fractions of Rac1 (Figure 1C) fluorescent staining revealed a Rac1 signal in the nuclei of both cell lines.

### 3.2 PI3K and multikinase inhibition promoted the disassembly of actin‐rich protrusions

**FIGURE 2:**
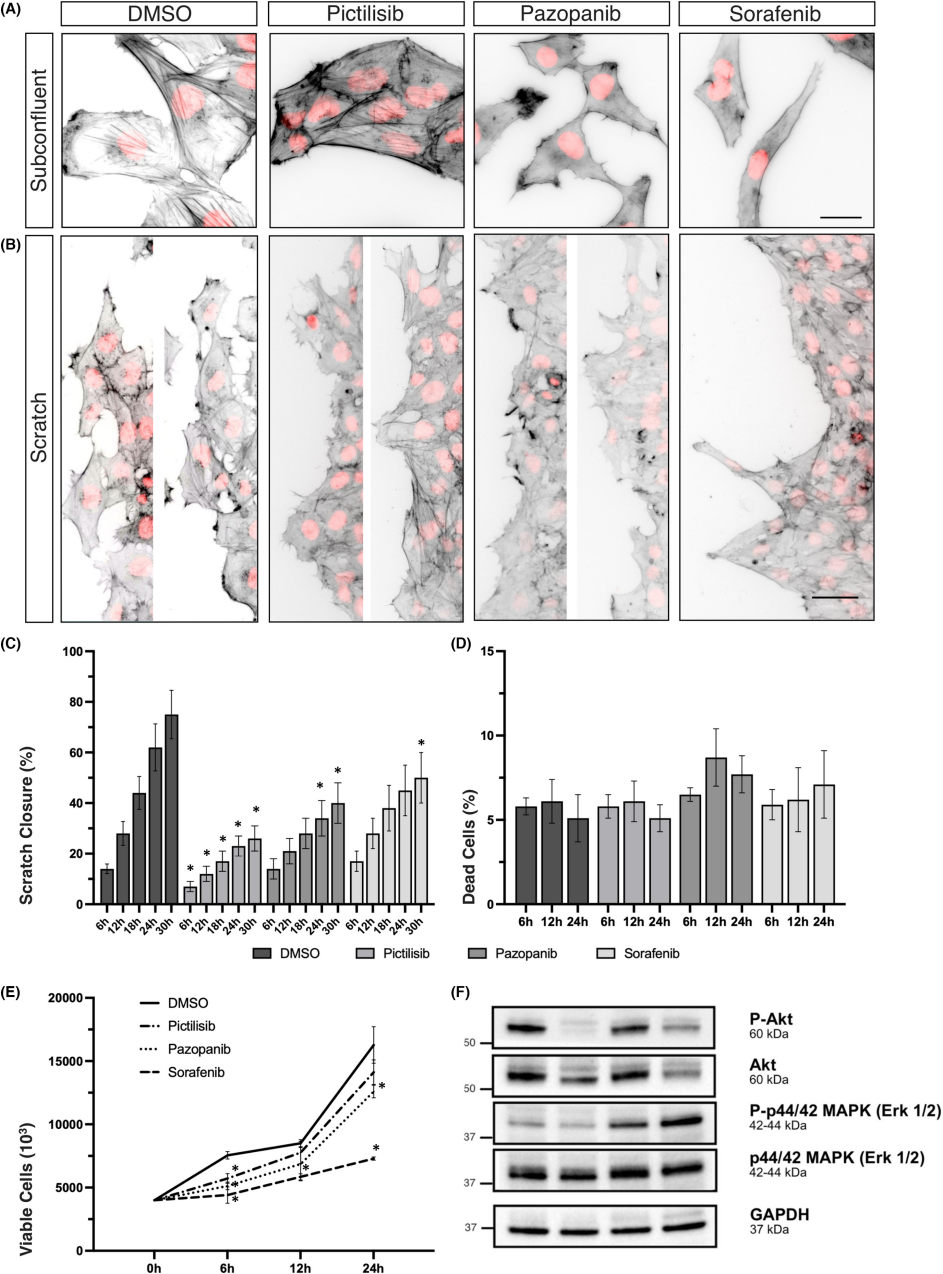
PI3K‐ and multikinase inhibition alter the actin cytoskeleton and interfere with migration of Daoy cells. (A) Daoy cells were seeded and Pictilisib (GDC‐0941, 1 μM), Pazopanib (15 μM), and Sorafenib (10 μM) were applied for 24 h. Cells were stained with Alexa Fluor‐555 coupled phalloidin to visualize the actin cytoskeleton (grayscale, inverted) and DAPI as nuclear counterstain (red). Images were captured using a fluorescence microscope (40x objective) and processed in ImageJ. Data are representative of two independent experiments. Scales indicate 25 μm. (B) A confluently grown monolayer of Daoy cells was scratch wounded, debris was gently washed off and fresh medium including kinase inhibitors was applied. After 24 h the actin cytoskeleton was stained with Alexa Fluor‐555 coupled phalloidin (grayscale, inverted) and DAPI as nuclear counterstain (red). Fluorescence microscopy was performed using a 10x objective. Two images of representative staining from two independent experiments are presented except for Sorafenib. In the latter we focused on showing a larger scratch border to better present elongated cells. Scales indicate 50 μm. (C) Scratches were performed as described above and treated with kinase inhibitors. After incubation for 0, 6, 12, 24, and 30 h non‐stained living cells were imaged using an inverted microscope in phase contrast taking care to image exactly the same scratch region. The kinetic of the scratch closure was analysed using NIS imaging software by measuring the size of the area of the cell free zone in each distinct scratch region after the indicated time interval. Results are shown in comparison to the untreated cells. All values below an asterisk show significantly lower scratch closure compared with the DMSO control group (*p < 0.05). The data represent the mean ± SD of two independent experiments. (D and E) Daoy cells were seeded in 6‐well plates and inhibitors were applied. The fraction of PI‐positive dead cells (D) and number of viable cells (E) was assessed after 6, 12 and 24 h by flow cytometry. All values below an asterisk show significantly changed cell survival compared with the DMSO control group (*p < 0.05). The data shown represent the mean ± SD of three independent experiments. (F) Daoy cells were treated with inhibitors for 24 h and cell lysates were prepared followed by SDS‐PAGE and immunoblot to assess total protein levels of (p‐)Akt and (p‐)Erk with GAPDH as loading control. The same GAPDH bands are shown in Figure 4B since they were part to the same experiment. The data shown are representative of two independent experiments.

**FIGURE 3:**
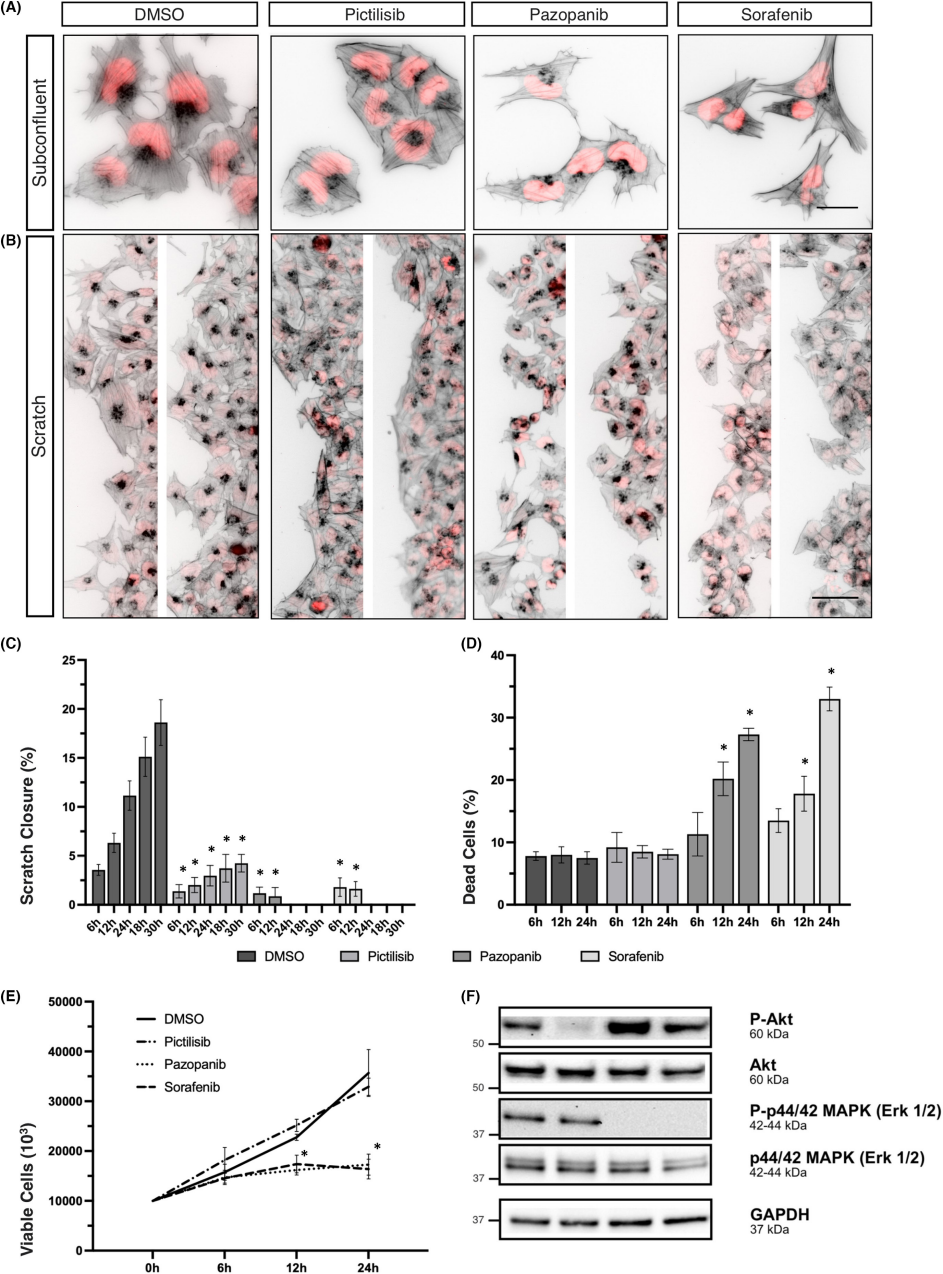
PI3K‐ and multikinase inhibition alter the actin cytoskeleton and interfere with migration of MEB‐Med‐8A. (A) MEB‐Med‐8A cells were seeded and treated with inhibitors for 24 h (Pictilisib) or 12 h (Pazopanib and Sorafenib) and stained with Alexa Fluor‐555 coupled phalloidin to visualize the actin cytoskeleton (grayscale, inverted) and DAPI as nuclear counterstain (red). Images were captured using a fluorescence microscope (40x objective) and processed in ImageJ. Data are representative of two independent experiments. Scales indicate 25 μm. (B) A confluently grown monolayer of MEB‐Med‐8A cells was scratch wounded, debris was gently washed off and fresh medium including kinase inhibitors was applied. After 24 h (DMSO and Pictilsib) and 12 h (Pazopanib and Sorafenib) the actin cytoskeleton was stained with Alexa Fluor‐555 coupled phalloidin (grayscale, inverted) and DAPI as nuclear counterstain (red). Fluorescence microscopy was performed using a 10x objective. Two images of representative staining from two independent experiments are presented. Scales indicate 50 μm. (C) Scratches were performed as described above and treated with kinase inhibitors. After incubation for 0, 6, 12, 24, and 30 h non‐stained living cells were imaged using an inverted microscope in phase contrast taking care to repetitively image the same scratch region. The kinetic of the scratch closure was analysed using NIS imaging software by measuring the size of the cell free area in the distinct scratch regions after the indicated time interval. Results are shown in comparison to the untreated cells. All values below an asterisk show significantly lower scratch closure compared with the DMSO control group (*p < 0.05). The data represent the mean ± SD of two independent experiments. (D and E) MEB‐Med‐8A cells were seeded in 6‐well plates and P3IK‐ or tyrosine kinase inhibitors were applied. The percentage of viable cells (E) and of PI‐positive dead cells (D) was assessed after 6, 12 and 24 h by flow cytometry. All values below an asterisk are significantly different from control (*p < 0.05). The data shown represent the mean ± SD of three independent experiments. (F) MEB‐Med‐8A cells were treated with inhibitors for 12 h and cell lysates were prepared followed by SDS‐PAGE and immunoblot to assess total protein levels of (p‐)Akt and (p‐)Erk with GAPDH as loading control. The same GAPDH bands are shown in Figure 4D since they were part to the same experiment. The data shown are representative of two independent experiments.

To examine the influence of the drug treatment on localization of Rac1 and cytoskeletal confirmation, microfilaments were fluorescently labelled by AlexaFluor‐555 coupled phalloidin (Figures 2A and 3A). Representative pictures after 24 h are shown except for MEB‐Med‐8A treated with Pazopanib and Sorafenib as the effect of these drugs was seen earlier and the MEB‐Med‐8A cells started to detach after 12 h of exposure to the MKI.

Application of Pictilisib resulted in the reduction of the cortical actin network and membrane protrusions. The cortical actin rich protrusions were thinning out in Daoy cells and both cell lines appeared in an overall more rounded shape. After 24 h of treatment with the PI3K inhibitor, cells of both cell lines were aggregated in small islands with rounded borders, tight cell–cell connections and more apparent stress fibres.

In contrast, Pazopanib and Sorafenib caused clumping of the microfilaments in the former filigree actin‐based protrusions, while the cells lost their rounded shape and the circular arrangement of actin, particularly in Daoy. The cellular appearance was more spindle‐shaped, as actin‐rich protrusions became elongated extending into different directions resulting in an overall stellate appearance of the cell formation. Cell–cell connections were less prominent and especially Sorafenib caused pronounced elongation in the cells in both cell lines. MEB‐Med‐8A cells progressively decreased in size and lost components of the cytoskeleton, which had spanned out the cytoplasm in untreated cells. Cell–cell connections were diminished, and cells arranged in a more linear manner. These effects were accompanied by increased detachment and ensuing apoptosis.

### 3.3 PI3K blockade by Pictilisib (GDC‐0941) and suppression of tyrosine kinase signalling by the MKI Pazopanib and Sorafenib caused cytoskeletal changes and interfered with migration

To evaluate the influence of PI3K‐ and multikinase inhibition on tumour cell mobility, we stained scratched cells with fluorescently labelled phalloidin and DAPI‐counterstain after DMSO or drug treatment. DMSO‐treated migrating Daoy cells presented hallmarks of collective cell migration in which one cell appeared to lead a group of cells (Daoy, Figure 2B). The leading cells displayed more actin‐rich protrusions than the cells that followed the leading edge. Untreated MEB‐Med‐8A cells exhibited a different migratory pattern and migrated more slowly into the scratch (MEB‐Med‐8A, Figure 3B). In contrast to Daoy cells, the MEB‐Med‐8A cells appeared not to migrate in group formation. They showed less actin‐rich protrusions of which just a few showed an orientation towards the opposite side of the scratch.

The application of the PI3K inhibitor Pictilisib led to an overall reduction of actin‐rich protrusions and flattened the scratch borders in both cell lines. In Daoy, it reduced the formation of groups reaching out into the scratch. These observations match with those made in the subconfluent cell staining (Figures 2A and 3A) and may well contribute to the impairment of migration in both cell lines. Treatment with the MKI Pazopanib and Sorafenib induced disassembly of actin‐rich protrusions at the scratch border in both cell lines and Sorafenib additionally led to an elongation of Daoy cells. In MEB‐Med‐8A, the MKI also caused an increasing loss of adhesive capacities resulting in the detachment of MEB‐Med‐8A cells after around 12 h of drug exposure.

### 3.4 PI3K inhibition impairs migration more effectively than interference with tyrosine kinase receptors

In order to compare the anti‐migratory capacity of Pictilisib, Pazopanib and Sorafenib, we analysed wound closure of the scratch assays with respect to early phase of drug exposure (Figure 2C (Daoy) and Figure 3C (MEB‐Med‐8A)). The PI3K inhibitor revealed the most effective inhibition of migration in Daoy cells. After 6 h, wound closure was already significantly impaired by Pictilisib. After 30 h of Pictilisib treatment, only 26.2 ± 4.6% of the scratch wound was closed compared with 74.8 ± 9.6% wound closure in untreated Daoy cells. The MKI slowed down the migration of Daoy cells less effectively with a significant anti‐inhibitory effect seen at 18 h following Pazopanib treatment. At that time, wound closure was at 27.6 ± 6.4% compared with 43.9 ± 6.5% in the untreated control. For Sorafenib, the inhibitory effect on migration reached significance only after 30 h of drug exposure with 50.2 ± 10.4% of the scratch closed compared with 74.8 ± 9.6% % in untreated cells. Sorafenib differed from Pazopanib and the PI3K inhibitor in that it also exerted considerable anti‐proliferative activity over the complete assessed time interval starting at 6 h of treatment (Figure 2E).

MEB‐Med‐8A cells migrate considerably slower than Daoy cells with only 18.62 ± 2.3% of wound closure in untreated MEB‐Med‐8A observed after 30 h (Figure 3C). As in Daoy, in MEB‐MED‐8A, the PI3K inhibitor suppressed cellular motility profoundly with significant attenuation of migration discernable as early as 6 h of Pictilisib application. Even after 30 h only 4.2 ± 0.7% of the scratch wound was closed. Of note, PI3K inhibition did not affect cellular survival or cellular proliferation and was therefore likely to dominantly suppress the migratory capacities during this early phase of drug exposure (Figure 3E).

Following treatment with the MKI, wound closure was observable only during the first 12 h. Thereafter, MEB‐Med‐8A lost their adhesive properties. This could be attributed to several effects. Profound morphological changes of the cytoskeleton as presented in Figures 2A and 3A was in part due to induction of MKI‐mediated apoptosis with 20% and 30% apoptosis at 12 and 24 h, respectively (Figure 3D). Still, particularly upon Sorafenib treatment, there was a threefold increase of the fraction of viable floating cells (n = 2; data not shown). Additionally, the anti‐proliferative activity of the MKI might have contributed to a delay in wound closure. In contrast to Daoy, in MEB‐Med‐8A cells, the lack of scratch wound closure was supposedly a cumulative effect of anti‐migratory, anti‐proliferative and pro‐apoptotic mechanisms. We additionally examined the influence of the substances on the phosphorylation of the signalling elements AKT and Erk 1/2 after 12 h (MEB‐Med‐8A, Figure 3F) and 24 h (Daoy, Figure 2F) of drug exposure. AKT phosphorylation was completely abrogated by PI3K inhibition in both cell lines without a distinct influence on Erk1/2 phosphorylation (Figure 3F). The treatment with the MKI resulted in upregulation of phosphorylated Erk 1/2 in Daoy cells in contrast to a complete abrogation of Erk 1/2 phosphorylation in MEB‐Med‐8A cells.

### 3.5 Levels of activated Rac1 were substantially reduced after treatment with Pictilisib, Pazopanib or Sorafenib

**FIGURE 4:**
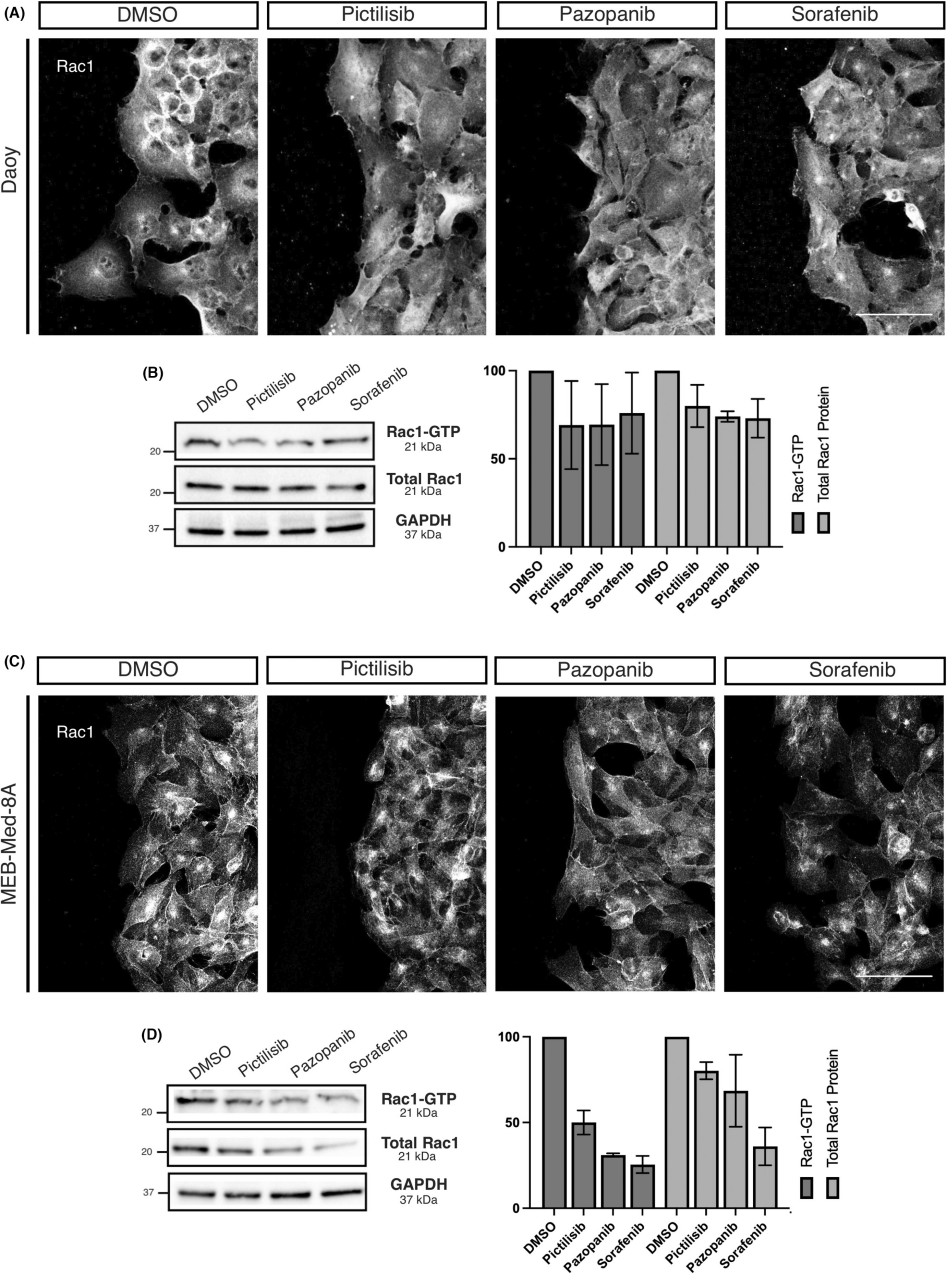
Levels of activated GTP‐bound Rac1 are reduced after PI3K blockage and inhibition of tyrosine kinases. (A and B). Confluently grown monolayers of Daoy or MEB‐Med‐8A cells were scratch wounded and treated with Pictilisib (GDC‐0941), Pazopanib or Sorafenib. After 12 h (MEB‐Med‐8A) or 24 h (Daoy) cells were fixed and stained with a Rac1 antibody to assess relative subcellular distribution of Rac1 at the scratch border. Scratch areas were imaged by confocal laser scanning microscopy using a 40x water immersion objective. Z‐stacks were performed to analyse Rac1 signals from top to bottom of the cells in 1024 × 1024 resolution using Galvano mode for the 488 laserline. Extended focus pictures were prepared using ImageJ software and show Rac1 distribution (grayscale) in the cells at the scratch border. Pictures presented are representative for two individual experiments. Scales indicate 50 μm. (B and D) Lysates of drug treated MEB‐Med‐8A (12 h) or Daoy (24 h) cells were prepared and a pulldown of GTP‐bound Rac1 was performed with a PAK PBD‐1 fusion protein followed by immunoblot analysis of Rac1. From the same lysates SDS‐PAGE followed by immunoblot to Rac1 were prepared to analyse the level of total cytoplasmatic Rac1. Densitometry analysis was performed using ImageJ 1.53c software. Signals of GTP‐bound Rac1 and total Rac1 levels were determined, and levels were compared with untreated control cells. Levels of total Rac1 were normalized to GAPDH as a loading control. The same GAPDH bands are shown in Figures 2F and 3F since the same blotted membranes were used in both experimental parts to determine expression levels of the respective proteins in relation to GAPDH of the same membrane. The data shown are representative of two independent experiments for the pulldown and four independent experiments for total cytoplasmatic Rac1 levels.

To examine the influence of the PI3K inhibitor and the MKI on the activity of Rac1, we performed a GTP‐Rac1 pulldown assay using a fusion protein corresponding to the p‐21 binding domain (PBD) of the p21‐activated kinase (PAK1) bound to a magnetic bead. PAK1 is a downstream effector which binds with the PBD in the Cdc42/Rac Interactive Binding Region (CRIB), when Rac1 is in an active GTP‐bound conformation. Activated Rac1 was reduced in both cell lines by treatment with either Pictilisib, Pazopanib or Sorafenib albeit to differing degrees (Figure 4B,D). In principle, reduction of activated Rac1 could be the result of reduced Rac1 protein or impaired conversion into its activated form. In MEB‐MED‐8A, we observed a reduction of Rac1 protein levels accompanied by a suppression of Rac1 activity in Pictilisib‐ and Pazopanib‐treated cells with a ratio of 0.62 and 0.45, respectively. Sorafenib treatment resulted in a profound reduction of Rac1 protein such that the ratio of activated to total Rac1 was 0.71 although as a net result activated Rac1 was markedly reduced to 25.5% in comparison with control. In Daoy, drug‐induced decrease of Rac1 protein level largely paralleled reduction of activated Rac1.

In addition to reduction of activated Rac1, all three drugs caused a visible effect on cytoplasmic Rac1 distribution (Figure 4A,C). In Daoy, demarcation of the nucleus by annular Rac1 staining became less pronounced and Rac1 was more spread throughout the entire cell. In MKI‐treated Daoy cells, change of cellular morphology to a more spindle‐shaped phenotype was accompanied by redistribution of Rac1. In MEB‐MED‐8A cells, perinuclear Rac1 accumulations were maintained following Pictisilib treatment with overall rarefication of Rac1 staining observed after MKI‐application (Figure 4C) which is in keeping with the Western blot results (Figure 4B,D).

## 4 DISCUSSION

Metastasis is the leading cause of death in children suffering from medulloblastoma and therefore a key target to further improve treatment strategies. The small Rho GTPase Rac1 has long been identified as a critical factor in migration, invasiveness, and metastasis in an array of cancers including medulloblastoma.^9^, ^20^, ^34^ Thus, Rac1 activation has been shown by genetic interference to drive medulloblastoma migration in vitro. Rac1 has been documented to be ubiquitously expressed among a wide range of tumour samples.^20^ Moreover, its direct downstream effector Pak1 has been found to be overexpressed in an association with unfavourable outcome.^9^ Beyond its pro‐migratory potential, Rac1 is a critical modulator of several other hallmarks of cancerogenesis such as tumour cell proliferation, anti‐apoptotic mechanisms, drug resistance and angiogenesis.^18^, ^19^ Upstream of Rac1, the PI3K/AKT pathway as well as PDGFR signalling, triggering the (RAF) RAS/MAPK/ERK cascade govern migration and proliferation in medulloblastoma.^2^, ^14^ These pathways have been linked to EMT promotion, a process by which cancer cells gain mesenchymal features and enhanced migratory capacity.^35^ Here, we document in two molecular types of medulloblastoma with high metastatic potential, activation of cytosolic Rac1, and pronounced phosphorylation of AKT and Erk1/2. Despite different subgroup affiliations and slightly different morphology, both exhibited mesenchymal features such as actin‐rich protrusions and loose cell connections. In consort, morphology and migratory pattern are slightly different, with Daoy cells migrating in groups and MEB‐Med‐8A cells moving more individually in keeping with findings reported for organotypic cerebellum slice cultures.^36^

Rac1 is known to be a key regulator in the multifaceted process of cancer cell migration and invasion, but irrespective of its pertinent role, clinically validated selective Rac1 inhibitors are not available to date.^19^, ^22^ We have previously shown that the MKI Pazopanib and Sorafenib, as well as the PI3K inhibitor Pictilisib, displayed promising anti‐neoplastic activity in vitro as well as in orthotopic xenograft models of medulloblastoma.^23^, ^24^ This alongside the clinical availability of the above‐mentioned MKI and the PI3K inhibitor formed the rationale to explore their potential for suppression of Rac1‐driven migration. Of note, activated Rac1 was reduced by PI3K inhibition as well as by the application of the MKI albeit to varying degrees. This is in line with our former observations that in both cell lines these MKI effectively suppress phosphorylated STAT3 which is recognized as a downstream effector of Rac1.^24^ The MKI however did not affect AKT‐phosphorylation despite downmodulation of GTP‐bound Rac1 particularly in MEB‐Med‐8A indicating that modulation of diverse signalling events can still merge in Rac1 inactivation as a common downstream event.

We show here that the reduction of GTP‐bound active form of Rac1 by the inhibition of PI3K is accompanied by impaired cellular migration in both cell lines associated with profound conformational cytoskeletal changes such as intensification of cellular connections and formation of isolated cellular clusters in the absence of any anti‐proliferative or pro‐apoptotic activity starting during the first hours of drug exposure. Concomitant to downmodulation of activated Rac1 by PI3K inhibition a loss of mesenchymal features can be observed.^35^ Consistent with their broad‐spectrum target range, MKI application provokes a more general breakdown of the actin cytoskeleton and disassembly of actin‐rich protrusions. The MKI are considerably less effective for impairing migration compared with direct PI3K‐blockade in Daoy. In MEB‐Med‐8A, MKI attenuate Rac1 activity and induce cellular detachment with a proportion of cells entering apoptosis. In this context, it is of interest that loss of cellular anchorage is known to trigger cellular death, a process called anoikis. Cell death caused by detachment requires the activation of several signalling pathways as well as intact p53.^37^ Daoy cells belong to a molecular type of medulloblastoma characterized by p53 mutations. This also fits with the reportedly central role of Erk1/2 in proliferation and migration, especially in SHH‐activated medulloblastoma to which the Daoy cell line belongs.^10^, ^15^, ^38^ Therefore, aberrant upregulation of Erk1/2 in Daoy cells in the presence of the MKI may serve as a cell survival signal which would explain the comparatively moderate anti‐proliferative effect of the MKI in Daoy cells while in MEB‐Med‐8A MKI‐mediated complete abrogation of Erk1/2 phosphorylation may also contribute to apoptosis.

Rac1 and Erk activity are interconnected in many cancer types and are reportedly subject to reciprocal regulation.^21^, ^32^, ^39^ Also, Erk phosphorylation may be modulated by the counter‐balancing effects of Rac1 and RhoA activity levels in medulloblastoma.^9^, ^20^ In addition to other influences, the PI3K pathway deserves consideration^20^, ^40^, ^41^ as Erk1/2 is situated in the midst of an intricately intertwined oncogenic signalling network. Paradoxical upregulation of Erk activation following a therapeutic interception of selected oncogenic pathways as observed here upon exposure to MKI is not uncommon and needs to be considered in targeted treatment design.^42^, ^43^, ^44^, ^45^ Thus, with the MAPK (RAF–MEK–ERK) signalling cascade facilitating cell proliferation, survival and transformation in a myriad of cancers, it is of concern that Raf inhibitors such as Sorafenib can induce a paradoxical activation of the MAPK pathway resulting in increased Erk1/2 phosphorylation, which has been linked to adverse effects.^46^ In a phase II study assessing the efficacy of Sorafenib in paediatric low‐grade astrocytoma, paradox upregulation of Erk phosphorylation was even associated with progressive tumour growth in a significant number of patients resulting in premature termination of the study.^44^ In medulloblastoma, following in vitro treatment with the MKI Dasatinib resulted in reduced mesenchymal migration via downregulation of ERK1/2 in the majority of cells; however, in a subset low‐level Erk1/2 activity was preserved. These cells acquired amoeboid morphology while maintaining invasive capacities.^15^ Although our 2D model system does not lend itself to definitively ascertain such a shift between mesenchymal and amoeboid migratory phenotypes, it is of note that Rac1 has been attributed a central regulatory role for these different modes of migration.^19^, ^47^

Besides its activation status, the spatio‐temporal localization of Rac1 determines its different cellular functions. At the leading edge, Rac1 drives cell migration through protrusion of lamellipodia while nuclear Rac1 influences are still subject to investigation and include modulation of the nuclear shape.^21^, ^33^ Here, we demonstrate that Rac1 is present in the nuclear and cytoplasmatic compartments in both medulloblastoma cell lines. Rac1 distribution is cell cycle dependent but can also be altered by phosphorylation through regulators including AKT and Erk1/2. This indicates that blockage of PI3K and tyrosine kinases potentially exerts a dual effect altering both Rac1 activity status as well as its localization.^21^ Rac1 phosphorylation at T108 is known to facilitate translocation of cytosolic Rac1 to the nucleus a process regulated by Erk activation.^32^, ^48^

Here, we show that PI3K inhibition as well as inhibition of tyrosine kinase driven signalling pathways in medulloblastoma cells decrease activated Rac1 particularly in MEB‐Med‐8A. In Daoy, redistribution of Rac1 is observed with dissipation of annular perinuclear Rac1 accumulation. In both untreated Daoy and MEB‐Med‐8A, we delineate for the first time localization of Rac1 in the nucleus. Aberrant localization of Rac1 to the nucleus has been reported to be involved in the organization of the nuclear membrane and results from an imbalance in nuclear Rac1 shuttling facilitated in part by the chaperone B23.^49^, ^50^ Nuclear accumulation of Rac1 has been reported to influence nuclear shape and plasticity associated with higher invasion capacities.^33^, ^49^ In MEB‐Med‐8A cells, deformed nuclei are particularly noticeable and present themselves kidney‐shaped with a prominent perinuclear actin aggregate co‐localizing with Rac1 in the nuclear indentation. Nuclear plasticity is a prerequisite for cancer cells to effectively navigate through the extracellular matrix.^51^

In this study, we illustrated the multifaceted role of Pictilisib, Pazopanib and Sorafenib as inhibitors of Rac1, as modulators of the cytoskeleton and migration in medulloblastoma. While significant progress has been made in designing Rac1 inhibitors for in vitro use, to date, there is no clinically approved inhibitor available.^19^, ^22^ Our results show that these clinically available therapeutics can suppress Rac1 activation and decrease migratory capacity in medulloblastoma. Indeed PI3K‐blockage is a potent means to induce a process resembling mesenchymal to epithelial transition (MET) and inhibit migration in medulloblastoma cells. Following inhibition of PI3K signalling, modulation of cytosolic Rac1 is associated with the reorganization of actin fibres and loss of the mesenchymal phenotype particularly in Daoy associated with a reduction in vimentin (data not shown). Also, tyrosine kinase receptors including PDGFR are promising therapeutic targets as they are expressed in primary medulloblastoma as well as metastases.^52^, ^53^ The morphological and molecular differences between the two high‐risk medulloblastoma cell lines and the differential impact of the tested drugs are exemplary for the challenges in targeted therapy of the different molecular subgroups in this highly malignant disease. Moreover, the intricate crosstalk between the two oncogenic PI3K/AKT and MAPK/ERK signalling cascades upregulated concomitantly not just in medulloblastoma but in a wide variety of cancer, warrant combined targeted approaches. This is of particular importance in view of the potential for paradox upregulation of individual elements following selective inhibition of one pathway alone. Combination strategies also address the issue of resistance following prolonged exposure to single targeted drugs. In medulloblastoma, we have already delineated enhanced anti‐neoplastic activity of PI3K inhibition with the MKI Axitinib and Vandetinib in vitro.^54^, ^55^ Whilst clinical trials evaluating combinations of specific inhibitors of the downstream effectors Erk1/2 and AKT exhibited considerable toxicity limiting clinical efficacy, the combination of Sorafenib and Pazopanib with Everolimus for PI3K inhibition seem to exhibit improved tolerability.^56^, ^57^, ^58^ Also has combined treatment with fibroblast growth factor receptor and PI3K inhibitors shown to increase anti‐proliferative capacities and enhance treatment sensitivity of medulloblastoma cells.^59^ These observations and the findings outlined here prospectively render a combined application of PI3K and multi kinase inhibitors as a promising therapeutic option targeting Rac1 as a key downstream effector governed by both signalling pathways in medulloblastoma.

## AUTHOR CONTRIBUTIONS

**Leonie Franziska Schön:** Conceptualization (equal); investigation (equal); visualization (lead); writing – original draft (lead); writing – review and editing (equal). **Rogerio Bastos Craveiro:** Conceptualization (equal); investigation (equal); methodology (equal); supervision (lead); writing – original draft (supporting); writing – review and editing (equal). **Torsten Pietsch:** Resources (equal); writing – review and editing (equal). **Thomas Moritz:** Writing – review and editing (equal). **Anja Troeger:** Conceptualization (supporting); writing – review and editing (equal). **Silvia Jordans:** Validation (supporting); writing – review and editing (equal). **Dagmar Dilloo:** Conceptualization (lead); methodology (equal); writing – review and editing (equal).

## CONFLICT OF INTEREST

The authors have no relevant financial or non‐financial interests to disclose. The authors declare that no funds, grants or other support were received during the preparation of this manuscript. The authors confirm that there are no conflicts of interest.

## Supporting information


**Table S1:** Antibodies used in western blot and immunocytochemistry

## Data Availability

The data that support the findings of this study are available from the corresponding author upon reasonable request.
