# Supplementary material for: The PI3K inhibitor pictilisib and the multikinase inhibitors pazopanib and sorafenib have an impact on Rac1 level and migration of medulloblastoma in vitro
Source: J Cell Mol Med. 2022 Nov 15;26(23):5832–45. doi: 10.1111/jcmm.17604 (PMC9716228; doi:10.1111/jcmm.17604)
Supplement: Supplementary file 1 — Table S1: Antibodies used in western blot and immunocytochemistry [file JCMM-26-5832-s001.pdf]

Supplementary Table 1: Antibodies used in western blot and immunocytochemistry

Antibody target	Antibody name/catalogue number	Supplier	Host / type of Antibody	Dilution
Rac1	Clone 102/Rac1 (RUO)	BD Biosciences	mouse, monoclonal	WB: 1:1000
	clone 23A8	Merck Millipore	mouse, monoclonal	ICC: 1:1000
AKT	Akt Antibody #9272	Cell Signaling	rabbit, polyclonal	WB: 1:1000
Phospho-AKT	Phospho-AKT (Ser473) (193H12) #4058	Cell Signaling	rabbit, monoclonal	WB: 1:1000
Erk1/2	p44/42 MAPK (Erk1/2) #9102	Cell Signaling	rabbit, monoclonal	WB: 1:1000
pERK1/2	Phospho-p44/42 MAPK (Erk1/2) (Thr202/Tyr204) #4370	Cell Signaling	rabbit, monoclonal	WB: 1:1000
GAPDH	GAPDH (D16H11) XP® #5174	Cell Signaling	rabbit, monoclonal	WB: 1:1000
Peroxidase-coupled anti-Rabbit	Goat IgG anti-Rabbit IgG (H+L)-HRPO, MinX Hu, Ms, Rt (#111-035-144)	Dianova	rabbit, polyclonal	WB: 1:10.000
Peroxidase-coupled anti-Mouse	Goat IgG anti-Mouse IgG (H+L)-HRPO, MinX Hu, Bo, Ho, Rb, Sw (#115-035-146)	Dianova	mouse, polyclonal	WB: 1:10.000
Eu-labeled anti-Rabbit	ScanLater goat anti-Rabbit Eu-labeled (#R8204)	Molecular Devices	goat, Europium-labeled	WB: 1:5000
Eu-labeled anti-Mouse	ScanLater goat anti-Mouse Eu-labeled (#R8205)	Molecular Devices	goat, Europium-labeled	WB: 1:5000
Alexa Fluor 488-labeled anti-mouse	Goat-anti mouse IgG (H+L) cross absorbed Alexa-Fluor Plus 488 (#A32723)	Invitrogen	goat, Alexa Fluor 488-labeled	ICC: 1:300
